# Supplementary material for: Smooth Interpolating Curves with Local Control and Monotone Alternating Curvature
Source: Comput Graph Forum. 2022 Oct 6;41(5):25–38. doi: 10.1111/cgf.14600 (PMC9827861; doi:10.1111/cgf.14600)
Supplement: Supplementary file 1 — Supplement Material [file CGF-41-25-s001.zip › Local-Smooth-Interpolating-MonoCurvature/extern/clothoids/docs/api-cpp/unabridged_orphan.html]

Full API — Clothoids v2.0.9

### Navigation

- index
- toc
- Clothoids »
- Full API

# Full API¶

## Directories¶

- Directory Clothoids
  - Files

## Files¶

- File AABBtree.cc
  - Definition (`AABBtree.cc`)
    - Program Listing for File AABBtree.cc
  - Includes
  - Namespaces

- File AABBtree.hxx
  - Definition (`Clothoids/AABBtree.hxx`)
    - Program Listing for File AABBtree.hxx
  - Included By
  - Namespaces
  - Classes
  - Functions

- File BaseCurve.hxx
  - Definition (`Clothoids/BaseCurve.hxx`)
    - Program Listing for File BaseCurve.hxx
  - Included By
  - Namespaces
  - Classes
  - Enums
  - Functions
  - Typedefs

- File BaseCurve\_using.hxx
  - Definition (`Clothoids/BaseCurve_using.hxx`)
    - Program Listing for File BaseCurve\_using.hxx
  - Included By

- File Biarc.cc
  - Definition (`Biarc.cc`)
    - Program Listing for File Biarc.cc
  - Includes
  - Namespaces
  - Functions

- File Biarc.hxx
  - Definition (`Clothoids/Biarc.hxx`)
    - Program Listing for File Biarc.hxx
  - Includes
  - Included By
  - Namespaces
  - Classes

- File BiarcList.cc
  - Definition (`BiarcList.cc`)
    - Program Listing for File BiarcList.cc
  - Includes
  - Namespaces
  - Functions

- File BiarcList.hxx
  - Definition (`Clothoids/BiarcList.hxx`)
    - Program Listing for File BiarcList.hxx
  - Includes
  - Included By
  - Namespaces
  - Classes

- File Circle.cc
  - Definition (`Circle.cc`)
    - Program Listing for File Circle.cc
  - Includes
  - Namespaces
  - Functions

- File Circle.hxx
  - Definition (`Clothoids/Circle.hxx`)
    - Program Listing for File Circle.hxx
  - Includes
  - Included By
  - Namespaces
  - Classes

- File Clothoid.cc
  - Definition (`Clothoid.cc`)
    - Program Listing for File Clothoid.cc
  - Includes
  - Namespaces
  - Functions

- File Clothoid.hxx
  - Definition (`Clothoids/Clothoid.hxx`)
    - Program Listing for File Clothoid.hxx
  - Includes
  - Included By
  - Namespaces
  - Classes

- File ClothoidAsyPlot.cc
  - Definition (`ClothoidAsyPlot.cc`)
    - Program Listing for File ClothoidAsyPlot.cc
  - Includes
  - Namespaces

- File ClothoidAsyPlot.hxx
  - Definition (`Clothoids/ClothoidAsyPlot.hxx`)
    - Program Listing for File ClothoidAsyPlot.hxx
  - Included By
  - Namespaces
  - Classes

- File ClothoidDistance.cc
  - Definition (`ClothoidDistance.cc`)
    - Program Listing for File ClothoidDistance.cc
  - Includes
  - Namespaces

- File ClothoidG2.cc
  - Definition (`ClothoidG2.cc`)
    - Program Listing for File ClothoidG2.cc
  - Includes
  - Namespaces
  - Functions

- File ClothoidList.cc
  - Definition (`ClothoidList.cc`)
    - Program Listing for File ClothoidList.cc
  - Includes
  - Namespaces
  - Functions

- File ClothoidList.hxx
  - Definition (`Clothoids/ClothoidList.hxx`)
    - Program Listing for File ClothoidList.hxx
  - Includes
  - Included By
  - Namespaces
  - Classes

- File Clothoids.hh
  - Definition (`Clothoids.hh`)
    - Program Listing for File Clothoids.hh
  - Includes
  - Included By
  - Namespaces
  - Defines
  - Typedefs

- File Fresnel.cc
  - Definition (`Fresnel.cc`)
    - Program Listing for File Fresnel.cc
  - Includes
  - Namespaces
  - Functions

- File Fresnel.hxx
  - Definition (`Clothoids/Fresnel.hxx`)
    - Program Listing for File Fresnel.hxx
  - Included By
  - Namespaces

- File G2lib.cc
  - Definition (`G2lib.cc`)
    - Program Listing for File G2lib.cc
  - Includes
  - Namespaces
  - Functions
  - Variables

- File G2lib.hxx
  - Definition (`Clothoids/G2lib.hxx`)
    - Program Listing for File G2lib.hxx
  - Included By
  - Namespaces
  - Classes
  - Functions

- File G2lib\_intersect.cc
  - Definition (`G2lib_intersect.cc`)
    - Program Listing for File G2lib\_intersect.cc
  - Includes
  - Namespaces
  - Functions

- File Line.cc
  - Definition (`Line.cc`)
    - Program Listing for File Line.cc
  - Includes
  - Namespaces
  - Functions

- File Line.hxx
  - Definition (`Clothoids/Line.hxx`)
    - Program Listing for File Line.hxx
  - Includes
  - Included By
  - Namespaces
  - Classes

- File PolyLine.cc
  - Definition (`PolyLine.cc`)
    - Program Listing for File PolyLine.cc
  - Includes
  - Namespaces
  - Functions

- File PolyLine.hxx
  - Definition (`Clothoids/PolyLine.hxx`)
    - Program Listing for File PolyLine.hxx
  - Included By
  - Namespaces
  - Classes

- File Triangle2D.cc
  - Definition (`Triangle2D.cc`)
    - Program Listing for File Triangle2D.cc
  - Includes
  - Namespaces
  - Functions

- File Triangle2D.hxx
  - Definition (`Clothoids/Triangle2D.hxx`)
    - Program Listing for File Triangle2D.hxx
  - Included By
  - Namespaces
  - Classes

### Quick search

### Table of Contents

- Matlab Interface Manual
- C++ API
- MATLAB API

«
hide menu

menu
sidebar
»

### Navigation

- index
- toc
- Clothoids »
- Full API

© Copyright 2021, Enrico Bertolazzi and Marco Frego.
Created using Sphinx 4.2.0.
